# Effects of Functional Acupuncture on Upper Limb Spasticity After Ischemic Stroke: A Protocol for a Randomized Controlled Parallel Clinical Trial

**DOI:** 10.3389/fneur.2022.835408

**Published:** 2022-05-18

**Authors:** Jinjin Mei, Yang Xue, Jingwen Li, Lihong Zhang, Jianyun Zhang, Yiying Wang, Kaiqi Su, Jing Gao, Jian Guo, Ruiqing Li

**Affiliations:** ^1^Henan University of Chinese Medicine, Zhengzhou, China; ^2^Rehabilitation Center, The First Affiliated Hospital of Henan University of Chinese Medicine, Zhengzhou, China

**Keywords:** upper limb spasticity, rehabilitation, functional acupuncture, motor function, ischemic stroke

## Abstract

**Background:**

Upper limb spasticity (ULS) is a common complication after stroke, which seriously affects the quality of life and rehabilitation of patients. There are different treatment methods for post-stroke spasticity (PSS). Our group found that functional acupuncture (FA) can effectively improve forearm spasticity and hand dysfunction after stroke, but the efficacy of ULS needs to be further verified. Therefore, this subject has mainly used clinical randomized controlled trials to evaluate the clinical efficacy of FA in the treatment of ULS after ischemic stroke.

**Method:**

This is a parallel design and randomized controlled trial. We selected 108 patients who met the predefined criteria and randomized them into two groups, the experimental group and the control group. The experimental group receives FA and routine rehabilitation treatment. The control group received traditional acupuncture (TA) and routine rehabilitation treatment. All patients received 20 courses of treatment for 4 weeks, and the modified Ashworth score (MAS), clinical neurological deficit score (CSS), Fugl-Meyer upper extremity function assessment (FMA-UE), and the Modified Barthel Index (MBI) scores were evaluated before and after treatment.

**Discussion:**

This trial is mainly to study the clinical efficacy of FA in the treatment of ULS after ischemic stroke. It will not only provide a new idea for the clinical treatment of upper limb post-stroke spasticity (ULPSS) but also will provide effective experimental support and a theoretical basis for the clinic.

**Trial registration:**

China Clinical Trials Registry No. ChiCTR2100050440. Registered on 27 August 27 2021.

## Introduction

Stroke is an acute or focal brain dysfunction caused by various vascular causes, such as hemorrhage and ischemia, with high incidence rate, disability rate, and mortality rate (1, 2). Ischemic stroke is the most common form, with 87% of all strokes (3). Limb spasticity is one of the common complications of a stroke, which seriously affects the functional recovery of patients, especially in the upper limbs (4). There is no clear definition of spasticity but is often defined as a motor disorder characterized by a velocity-dependent increase in tonic stretch reflexes after upper motor neuron syndrome (5, 6). The upper limb projection area accounts for a large proportion in the cerebral cortex, there can be more obvious functional damage to the upper limb after the lesion, so the upper limb spasticity (ULS) is more serious than that of the lower limb (7). The ULS may cause problems related to (1) passive function (e.g., wearing, eating, and cleaning hands); (2) pain; and (3) muscle and joint contractures (8). It can directly affect the quality of daily life of patients, the difficulty of recovery is significantly higher than that of lower limbs, and it can bring heavy pressure and economic burden to the patient's family and society (9–11). Therefore, it has a wide range of clinical significance to find an effective treatment for upper limb post-stroke spasticity (ULPSS).

In recent years, more and more attention has been paid to the issue of spasticity. At present, western medicine has no specific and systematic treatment method for ULPSS. It mainly conducts rehabilitation training, such as physical therapy, drug therapy, botulinum toxin A (BoNT-A), and external mechanical adjuvant therapy (12–14). However, each traditional treatment method has its limitations. There is no fixed standard for the parameters and stimulation intensity of physical therapy, which only depends on personal preference and experience (15). Although drug therapy has obvious effects, it is prone to side effects, such as sleepiness, fatigue, nausea, and muscle weakness (16). Long-term injection of BoNT-A may cause local pain, muscle weakness, edema at the injection site, and other adverse reactions and cannot significantly improve the quality of life and limb function of patients with post-stroke spasticity (PSS) (17). Traditional Chinese Medicine (TCM) treatment focuses on acupuncture and moxibustion, massage, Chinese medicine rehabilitation nursing, hot compress, and external application of Chinese medicine, which has a good curative effect and is widely used (18–20). At present, many studies have shown that TCM is very effective in the treatment of stroke hemiplegia with acupuncture. Stimulating acupoints through acupuncture can stimulate brain cells to activate after stimulation and promote the establishment of cerebrovascular collateral circulation in patients. It has been widely used in the treatment of PSS (21, 22). In addition, the results of clinical trials and meta-analysis show that acupuncture can significantly improve the grade of spasticity, help patients to restore muscle tension and motor function, improve the quality of life, and reduce the disability rate (23). Because of its unique advantages, such as significant curative effect, low price, and small side effects, it is favored by more and more clinicians and patients and results in a lot of innovative acupuncture methods.

Our group through reviewing the literature and combining with a summary of clinical practice experience found that the use of functional acupuncture (FA) can effectively improve forearm spasticity and hand dysfunction after stroke (24–26), but the efficacy of ULS needs to be further verified. FA is a new acupuncture method that combines traditional acupuncture (TA) with modern rehabilitation theory and selects two common acupoints and two functional points to maximize the recovery of patients' various functions (27). FA can enhance sensory transmission, simplify acupoints, and have a great effect. It can be divided into three steps: rapid needling of the acupoint Daling (PC7), penetrating needling of the acupoint Hegu (LI4), and electrical stimulation of the functional acupoint. After rapid needling at PC7, the contracture fingers can be relaxed to a state of no flexion, passive activity, and no resistance and can last for several hours (28). The penetrating needling of LI4 to Houxi (SI3) can directly alleviate the spasticity of finger flexor muscle and indirectly excite and stretch the extensor tendon of a finger. By repeatedly stimulating the flexor tendon, the spasticity can be alleviated and the effect of improving finger dysfunction can be achieved (18, 29). In this state, the use of functional acupoint electrical stimulation can quickly improve the overall function of the upper limb. Then this topic is mainly to study the clinical efficacy of FA in the treatment of ULS after ischemic stroke.

In recent years, clinical experiments and basic research have found that acupuncture has an obvious therapeutic effect on ULPSS, it is considered by the World Health Organization (WHO) as an alternative and complementary strategy for the treatment and improvement of stroke (23), but the efficacy differences between different acupuncture methods are uncertain. Few trials involve the comparison of different acupuncture methods to verify the efficacy differences between different acupuncture interventions. In this study, FA was used to treat ULS after ischemic stroke, verifying the efficacy and rehabilitation significance of FA, which makes up for the study on the lack of different acupuncture methods in this disease, enriching the treatment of the ULSPSS, and providing a reference for clinical treatment of this disease.

## Methods and Analysis

### Trial Design and Setting

**Figure 1:**
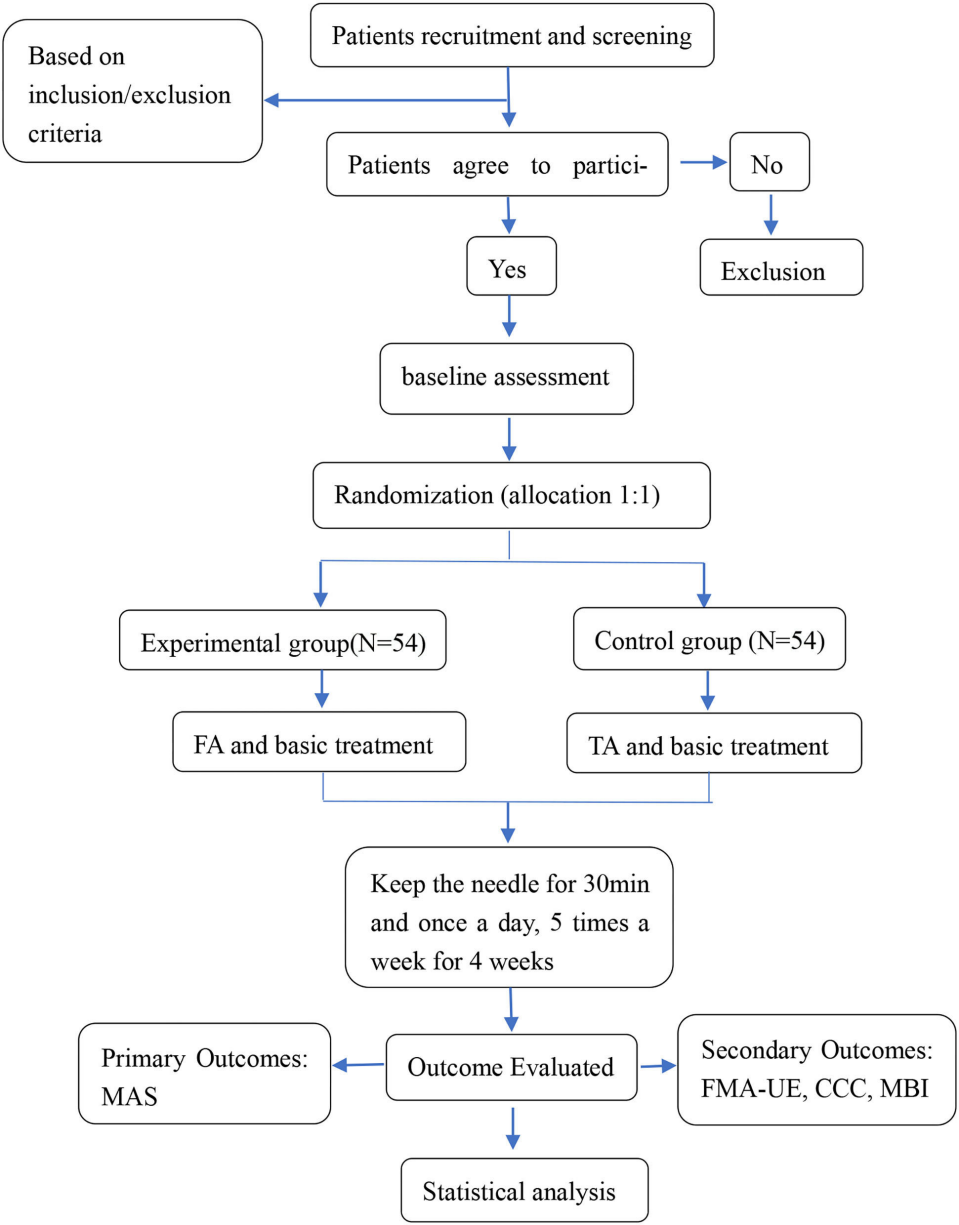
Flowchart of the trial. FA, functional acupuncture; TA, traditional acupuncture; MAS, the Modified Ashworth Scale; FMA-UE, the Fugl-Meyer Assessment for Upper Extremity; CCC, clinical neurological deficit score; MBI, the Modified Barthel Index.

This was a parallel design and randomized controlled trial. The subjects were be recruited from the Rehabilitation Center of the First Affiliated Hospital of Henan University of Chinese medicine. We selected 108 patients who met the predefined criteria and randomly divided them into 2 groups, with 54 patients in each group: the experimental group and the control group. All patients received 20 courses of treatment for 4 weeks, and the upper limb function was evaluated before and after treatment. The efficacy of the experimental group when compared with the control group was analyzed after data collection. An example template for the content of admission plans, interventions, and evaluations is shown in Table 1. Our protocol presentation is in accordance with the Standard Protocol Items: Recommendations for Interventional Trials 2013 (SPIRIT 2013) guidelines (30). The study flowchart is presented in Figure 1.

**Table 1:** Qualification screening and informed consent was completed before the assignment.

**Timepoint**	**Enrollment**	**Allocation**	**Treatment period**			
	**−1 week**	**0 week**	**1 week**	**2 week**	**3 week**	**4 week**
Enrollment						
Eligibility screen	X					
Baseline	X					
Informed consent	X					
Medical history	X					
Merger disease	X					
Allocation		X				
Interventions						
Experiment group			X	X	X	X
Control group			X	X	X	X
Assessments						
MAS		X				X
FMA-UE		X				X
CSS		X				X
MBI		X				X
Safety evaluation		X				X
Needle sensation			X	X	X	X
Adverse events			X	X	X	X

*After allocation, each patient was treated within 4 weeks. The clinical outcome was evaluated twice: after allocation and after treatment. Adverse events were recorded on the case report form (CRF) at any time during treatment*.

### Randomization

Eligible patients were allocated, in a 1:1 ratio were randomly assigned to the experimental group or the control group, following the randomization principle, which employs block randomization to generate random-number sequence using the SPSS, and an independent statistician who did not participate in the treatment and result evaluation was responsible for processing the results. Meanwhile, we also prepared sealed opaque envelopes with group information strips in these envelopes, which was hidden until the informed consent was obtained.

### Blinding

Due to the limitations of this protocol and the nature of acupuncture treatment, the acupuncturists participating in this trial could not be blinded to the assignments, the patients were blinded. Patients were informed that they will be randomized to receive one of two effective interventions after registration. Every patient knew the type of treatment they received, but they did not know another type. Each patient's acupuncture intervention was carried out in a separate personal space and at different times to avoid communication between patients. The distribution of patient groups by data managers and statisticians was not clear. Experts, data managers, and statisticians were not allowed to communicate with others about patients' treatment, so as to improve the accuracy of test indicators and ensure the effectiveness of interventions and the objectivity and reliability of the evaluation. The blind method was made public after the statistical analysis was completed.

### Inclusion and Exclusion Criteria

Inclusion Criteria. The Eligible Participants Should Meet all of the Following Criteria:

(1) The patients met the diagnostic criteria of ischemic stroke and were diagnosed by computed tomography (CT) or magnetic resonance imaging (MRI).

(2) Patients with clinical symptoms of paralysis on one side of the limb and upper extremity motor dysfunction.

(3) Patients with the MAS is the grade I and above, grade III and below.

(4) Men or women with an age range between 20 and 70 years.

(5) The Brunnstrom recovery stage (BRS) of the patient's upper extremity ranged from II to IV.

(6) Patients who are stable, conscious, and have no communication problems.

(7) Patients with no previous history of upper extremity dysfunction, first onset, or legacy of stroke.

(8) The participants were voluntary and signed the relevant informed consent.

Exclusion Criteria. Criteria for Exclusion Are the Following:

(1) Patients with upper extremity motor dysfunction, combined with severe cardiovascular disease, hypertension, respiratory infection, severe renal dysfunction, severe diabetes mellitus, and a history of psychiatric, were accompanied by agnosia, apraxia, and an intellectual disability that affects communication and evaluation.

(2) Patients with unstable vital signs in the acute phase.

(3) Patients in thrombolytic therapy.

(4) Fear of acupuncture and poor physical fitness, such as easy to faint, are not suitable for acupuncture treatment of patients.

(5) Patients who have taken sedative drugs and muscle relaxants in the recent past.

(6) Patients with transient ischemic attack (TIA), reversible ischemic neurological deficits, subarachnoid hemorrhage, etc.

(7) Patients who are pregnant, lactating, or preparing for pregnancy.

#### Elimination Criteria

(1) Patients who do not meet the criteria for a diagnosis of ULPSS and are mistakenly included.

(2) Patients who have serious adverse events (SAEs), special physiological changes and complications, and should not continue the test.

### Sample Size

The purpose of this study was to evaluate the efficacy of FA in the treatment of ULPSS. According to previous research and clinical experience, the effective rate of TA is about 65%, and the effective rate of FA will probably reach 85% (31). The significance test level is 0.05, and the test power is 0.9. The sample size is calculated by using:


$$\begin{array}{l} N=P\left(1-P\right){\left(\frac{\alpha +\beta }{\delta }\right)}^{2} \end{array}$$

where N is the required sample size for each treatment group, and the sample size of each group is equal. When α is 0.05 and β is 0.1, the normal distribution quantile table shows that:


$$\begin{array}{l} Z_{\text{α}/2}=1:645 \end{array}$$

and


$$\begin{array}{l} Z_{\text{β}/2}=1:282 \end{array}$$

P_0_ and P_1_ represent the original curative effect and the expected curative effect, 65 and 85%, respectively. δ represents the mean difference between two groups, by substituting the above parameters and values into the formulas, 49 cases will be needed for each group. Accounting for a 10% expulsion rate, the final estimated sample size will be about 54 cases per group (108 in total).

### Recruitment

We had formulated plans and methods related to subjects, such as preparing informed consent, and obtaining necessary data, videos, and pictures to help subjects to understand the purpose and procedure of the trial. We had also explained the advantages and disadvantages of this treatment and relevant safety measures to be taken during the trial.

In addition, we had put up recruitment posters in hospitals and community billboards and used the Internet for publicity and recruitment. The research group had conducted preliminary judgment and screening on each participant according to the above inclusion and exclusion criteria, conducted a final review after signing the informed consent, and those who passed the review were included in the test.

### Interventions

The experiment was divided into two groups: the experimental group and the control group. The two groups of patients received different treatments five times a week for 4 weeks. At the same time, each patient received symptomatic treatment and supportive treatment. On the basis of routine rehabilitation treatment, acupuncture was used in the groups. The experimental group was treated with FA, and the control group was treated with TA.

#### Experimental Group

Acupoints used were Daling (PC7), Hegu (LI4), functional acupoint 1, and functional acupoint 2. The locations are specifically presented in Table 2. Operation: Sterile acupuncture needles (single tube needles) of 0.30 mm × 75 mm, 0.30 mm × 30 mm were used (produced by Wuxi Jiajian Medical Instruments Co., Ltd.). Routine disinfection of the acupuncture site, Step 1, quick puncture PC7: we used 30 mm × 30 mm one-time filigree needle to quickly pierce in the direction of the palm and heel. After “Deqi,” repeatedly lifted and twisted the needle. The twisting frequency was 100 times/min, and the lifting time was 30 s. There was no need to leave a needle after obtaining a strong needle sensation in the local tissue (touching the inductance to the fingertip). Step 2, 0.30 × 75 mm filiform needle was punctured 50~60 mm from LI4 to SI3 direction. After “Deqi,” it was repeatedly lifted, inserted, and twisted. The twisting frequency was 60 times/min and the lifting and inserting time was 1 min. The needle was retained after the strong needle feeling of deep tissue was obtained and the finger spasm was relieved immediately. Step 3: Electrical stimulation of functional points: we used g6805-a electroacupuncture instrument at functional point 1 and functional point 2, density wave; Negative pole was connected to functional point 1 and positive pole was connected to functional point 2. The pulse current was stopped when the patient had slight muscle contraction. We gently adjusted the direction and depth of the needle tip of functional point 1, so that the five fingers of the affected hand was extended at the same time and the back of the wrist was extended. We kept the needle for 30 min. The treatment was performed once a day, 5 times a week for 4 weeks. The curative effects were evaluated before and after treatment.

**Table 2:** Location of acupoints for treating post-stroke upper limb spasticity.

	**Acupoints location**
Daling (PC7)	Located at the midpoint of the transverse stripes of the palm of the hand, between the palmar longus tendon and the flexor carpi radialis tendon.
Hegu (LI4)	Located between the first and second metacarpal bones on the back of the hand, at the midpoint of the radial side of the second metacarpal bone.
Functional point 1	Location roughly equivalent to the position of the elbow-liu (LI12) point in the Large Intestine meridian of the hand
Functional point 2	Location roughly equivalent to the location of the Waiguan (SJ5) in the triple energizer meridian of the hand.

#### Control Group

Acupoints used were Jianyu (LI15), Quchi (LI11), Shousanli (LI10), Waiguan (SJ5), Hegu (LI4), and Baxie (EX-UE9). Operation: Sterile acupuncture needles of (single tube needles) 0.30 mm × 40 mm milli-needles (produced by Wuxi Jiajian Medical Instruments Co., Ltd.) were used. The conventional straight puncture method was used. Routine disinfection of acupuncture site, direct puncture with a 0.30 × 40 mm filiform needle, 15~25 mm depth of penetration, and LI10 and LI4 were connected to the G6805-a electroacupuncture instrument, sparse and dense waves, the intensity is as tolerated by the patient, and then the needle is retained for 30 minutes. The treatment was performed once a day, 5 times a week for 4 weeks. The curative effects were evaluated before and after treatment.

### Outcome Assessments

All the data collectors received strict professional training to assess the degree of clinical neurological deficits and upper extremity motor function of the patients before treatment, after one course of treatment and after three courses of treatment and to fill in the modified Ashworth score (MAS), clinical neurological deficit score (CSS), Fugl-Meyer upper extremity function assessment (FMA-UE), and the Modified Barthel Index (MBI) score to established case experimental forms of patients.

#### Primary Outcomes

The main result is the MAS, which is the most widely used clinical spasm scale (32). It is usually used to obtain the baseline evaluation of increased muscle tone, monitor the course of the disease, determine the effectiveness of drugs, and rehabilitation interventions, so as to reduce the overall increase of muscle tension, normalize the muscle tone of the selected muscle group, and guide physical therapy and other treatment decisions. It has the advantages of simplicity, time saving, and convenient operation. It is divided into 6 levels, i.e., 0, I, I +, II, III, and IV. Scoring methods, i.e., 0, 1, 2, 3, 4, and 5 points, represent 0, I, I +, II, III, and IV, respectively. The higher the MAS score, the more serious the spasticity. Compared with the lower limbs, the reliability of the upper limbs is better (33). The specific contents of MAS are shown in Table 3.

**Table 3:** The modified Ashworth score (MAS)-specific scoring rules.

	**Modified Ashworth Scale**
0	No increase in muscle tone
1	Slight increase in muscle tone, manifested by a catch or by minimal resistance at the end of the range of motion (ROM) when the affected part(s) is (are) moved in flexion or extension
1+	Slight increase in muscle tone, manifested by a catch, followed by minimal resistance throughout the remainder (less than half) of the ROM
2	More marked increase in muscle tone through most of the ROM, but affected part(s) easily moved
3	Considerable increase in muscle tone, passive movement difficult
4	Affected part(s) rigid in flexion or extension

#### Secondary Outcomes

The FMA-UE, CSS, and MBI were secondary outcome indicators.

The FMA-UE is often a widely used evaluation tool for evaluating upper extremity motor function during stroke rehabilitation. It can effectively, stably, and sensitively reflect the changes in patients' dysfunction with good reliability and validity (34, 35). Evaluate the flexion, coordination, range of motion, stability, and coordination of each joint of the patient's upper limb, which is composed of 33 items in four parts: hand, wrist, elbow, and shoulder, with a total score of 66. The lower the score, the more serious the upper extremity dysfunction. The minimum clinically important difference (MCID) of FMA-UE was 4.25–7.25 points (36). That is, if the difference in upper extremity motor function score before and after treatment is >MCID, it is considered that the upper extremity motor function of the patient has been improved, the treatment is effective and can surpass the random error (37).

The CSS was determined by the “Fourth National Conference on Cerebrovascular Diseases” (1995), which is mainly to evaluate the curative effect and is divided into basic cure (the score of functional defect was decreased by 91–100%, and the degree of disability was grade 0), significant effect (the score of functional defect was decreased by 46–90%, and the degree of disability was grade 1), improvement (the functional defect score was decreased by 18–45%), and ineffective (the functional defect score decreased by about 17%). The score is simple and easy to use (38).

The MBI is a standard scale that is widely used to evaluate the dysfunction of basic activities of daily living, which consists of 10 daily life activities, such as bathing, toilet, eating, and dressing, with a total score of 100. The higher the score, the better the self-care ability in daily life (39).

### Safety Evaluation and Adverse Events

During the whole treatment process, any adverse events and their treatment methods will be recorded. Adverse events associated with acupuncture treatment included severe pain, syncope, bleeding, or any other discomfort. During the intervention, researchers will pay close attention to the patient's condition. If the patient has any discomfort, the intervention will be stopped immediately and the patient's situation will be handled accordingly. Details of any medical malpractice will be reported in detail in the CRF. All adverse events will be regularly reviewed by the lead investigator, and adverse events that meet the criteria for SAEs will be reported to the local institutional review committee.

### Data Collection and Management

The data of all patients will be truthfully, accurately, and timely recorded on the CRF, such as experimental time point, result measurement, adverse events, and safety evaluation. Two independent researchers input data and keep the participants' personal information strictly confidential. The data will be safely saved by our data researchers and monitored by the ethics committee of the First Affiliated Hospital of traditional Chinese medicine of Henan University, as shown in Table 1. At the end of the test, study participants shall submit CRF in time and submit a test summary as required.

### Statistical Analysis

After all the experimental data are verified to be correct, SPSS19.0 statistical software is used for statistical analysis. Classified variables will be analyzed using Pearson's χ^2^ test or Fisher's exact test, and continuous variables will be evaluated using Student's *t*-test or an appropriate non-parametric method. The statistical results are measured by the mean ± standard deviation (SD; *x* ± s); when comparing within or between groups, tests for normality and homogeneity of variance are performed prior to data analysis for each group. If the data satisfy normal distribution and homogeneity of variance, the *t*-test will be used for comparisons between two groups, and the least significant different (LSD) or Student-Newman-Keuls (SNK) method will be used for multiple comparisons. Instead, the rank-sum test will be used for non-normality or non-uniformity of variance. Set the test level as 0.05, *p* < 0.05, and the difference was statistically significant.

### Quality Control

In order to reduce potential errors and ensure the quality of the trial, all acupuncturists and evaluators must receive professional training before the trial. The training course includes the recruitment, intervention, and evaluation processes. The integrity and accuracy of the data will be monitored by clinical trial experts from the clinical research center of the First Affiliated Hospital of traditional Chinese medicine of Henan University. All clinical trial experts are independent of the subject principle. In addition, we will set up a quality control group to supervise whether the experimental procedures comply with the standard guidelines. When the total number of samples collected reaches 54, statistical analysis will be carried out. The lead investigator will obtain these interim results and decide whether to continue the trial. If the efficacy of the experimental group is lower than that of the other two groups in the results of interim data, we will stop the trial.

## Discussion

Spasticity is one of the most common causes of physical disability in the world. Patients often recover their lower limb function faster in rehabilitation training, while the upper extremity function, especially the fine motor function of the hand, usually recovers for a long time, which seriously affects the daily life of patients (40). In recent years, the achievements of modern rehabilitation medicine and acupuncture mechanism have made acupuncture and moxibustion more effective in treating ULPSS, improving clinical symptoms of ULPSS, preventing cerebrovascular diseases, and reducing the incidence rate of PSS, but some problems still need to be solved. The purpose of this study was to evaluate the efficacy and safety of FA in the treatment of ULPSS.

### Selection of Acupoint Prescription

In this study, FA was selected, and its acupoint selection was also exquisite. It mainly includes PC7, LI4, and functional point. PC7 (41) is an important point of the pericardium meridian (PC). It is on the palmar side of the forearm, between the palmar long tendon and the radial wrist flexor tendon. The tissues involved after rapid acupuncture include palmar long tendon and radial wrist flexor tendon, internal and external cutaneous nerves, median nerve trunk, and interosseous nerve of forearm, which can relax the contracture hand fingers to a state of no flexion, passive activity, and no resistance for several hours. LI4 (42) is the original point of the large intestine meridian (LI), and the LI reaches the head. Stimulating this point can make the Jing Qi (essence) reach the head, regulate the health with the camp, calm Yin and Yang, and calm the brain and mind. Acupuncture emphasizes that acupuncture “leaves acupoints and does not leave meridians” (43). Acupuncture combines Yin and Yang, dredges meridians, dredges Jing Qi, regulates blood and Qi, and connects multiple meridians. The penetrating needling of LI4 to SI3 can directly alleviate the finger flexor spasticity and indirectly excite and stretch the finger extensor tendon. By repeatedly stimulating the flexor tendon, it can alleviate the spasticity and improve the finger dysfunction. In this state, the use of functional point electrical stimulation can quickly improve the overall function of the upper limb. Functional points 1 and 2 are two new points found in the process of clinical treatment. The treatment method we used for these two points is called “FA.” The body surface positioning of functional points 1 and 2 is not fixed. Functional point 1 is roughly equivalent to the position of Zhoumiu (LI12) of the Large Intestine meridian (LI). There is a radial neuromuscular junction under it. Stimulating this point can produce finger extension and wrist dorsum flexion. In actual operation, when the electroacupuncture instrument is connected, the adjustment of the needle tip direction to produce the maximum passive action with the minimum current intensity is the key to the FA. At this time, the electroacupuncture instrument and the connected electroacupuncture can achieve the function of functional electrical stimulation, but its effect is better than the functional electrical stimulation with the surface electrode as the contact point; Functional point 2 is roughly equivalent to the position of Waiguan (SJ5) point of the triple energizer (TE) meridian. As an auxiliary point, it is connected to the positive pole of the electroacupuncture instrument. It can be seen from the above that this is an innovative acupuncture method with a unique acupoint selection. It adopts the combination of traditional acupoints and anatomical position, the combination of traditional needling method and through needling and fast needling, and the combination of traditional needling feeling and strong needling feeling. There are few clinical studies. This study selects TA as the control, selects the commonly used acupoints of ULS from data mining technology for comparison, which can intuitively feel the clinical efficacy of this acupuncture method.

### The Combination of Subjective and Objective Outcome Measures

In addition to the scale evaluation selected in this study, we also adopted the evaluation methods, such as surface electromyography (sEMG) and electrophysiological measurement (H-reflex), and adopted a combination of subjective and objective methods to reduce the impact of a series of human interventions on the experimental results and make the study more convincing (44).

One important limitation of our study is an acupuncture trial, it is impossible to perform a double-blind procedure. In order to reduce this bias, each group of patients will receive treatment at different times in different rooms, and communication between each group of patients is prohibited. In addition, all acupuncturists participating in this study will receive unified training, strictly standardize acupuncture operation, and eliminate the error of the study to the greatest extent.

In conclusion, this report describes a randomized controlled trial that systematically evaluates the rehabilitation effect of FA on patients with ULPSS. To observe the clinical effect of functional acupuncture on ULPSS, and to determine whether FA can effectively improve the ULS and improve the extremity motor function and self-care ability of patients after stroke. This study not only provides a new idea for the clinical treatment of ULPSS but also provides effective experimental support and a theoretical basis for the clinic.

### Trial Status

The recruitment of patients for this study began on 1 September 2021, and this study is ongoing. Protocol version number and date: V2.0, 1 September 2021. The recruitment of patients is expected to be completed in May 2022.

## Ethics Statement

The studies involving human participants were reviewed and approved by the Ethics Committee of the First Affiliated Hospital of Henan University of Chinese Medicine. The patients/participants provided their written informed consent to participate in this study.

## Author Contributions

RL and YX proposed the concept for this trial and designed the study. JM and YX contributed equally to the conception, design, and manuscript writing. LZ, JZ, YW, and JGao helped search the literature and assisted in the recruitment of patients. JL, KS, and JGuo participated in the revision and editing of this manuscript. All the authors approved the final version of the manuscript.

## Funding

This study was supported by Henan Provincial Health Commission, China (no. 2019JDZX2065) and Henan Province Chinese medicine scientific research special topic, China (no. 2019ZY2129). Henan Provincial Higher Education Key Research Project Plan, China (no. 21A360023). These study funders have no role in the study design, data collection and management, and manuscript writing publication.

## Conflict of Interest

The authors declare that the research was conducted in the absence of any commercial or financial relationships that could be construed as a potential conflict of interest.

## Publisher's Note

All claims expressed in this article are solely those of the authors and do not necessarily represent those of their affiliated organizations, or those of the publisher, the editors and the reviewers. Any product that may be evaluated in this article, or claim that may be made by its manufacturer, is not guaranteed or endorsed by the publisher.

## Abbreviations

ULS: Upper limb spasticity
ULPSS: upper limb post-stroke spasticity
PSS: post-stroke spasticity
FA: Functional Acupuncture
TA: Traditional Acupuncture
BoNT-A: botulinum toxin A
SPIRIT, Standard Protocol Items: Recommendations for Interventional Trials
CT: Computed tomography
MRI: Magnetic resonance imaging
PASS: Power analysis and sample size
SPSS: Statistical Package for the Social Sciences
RMS: Root mean square
MAS: Modified Ashworth Scale
FMA-UE: Fugl–Meyer Assessment for Upper Extremity
MBI: Modified Barthel Index
CRFs: Case report forms
sEMG: Surface electromyography
H-reflex: electrophysiological measurement.
